# Therapeutic vaccination with the Ag85B-Rv2660c-MPT70 fusion protein enhances *Mycobacterium tuberculosis* H37Ra clearance in post-exposure mice

**DOI:** 10.3389/fimmu.2025.1624923

**Published:** 2025-08-14

**Authors:** Zhiming Hu, Shaohua Guo, Wenlong Chen, Jiangshan Ouyang, Chunxu Huang, Ting Cao, Jun Mou, Xinxia Gu, Jie Liu

**Affiliations:** ^1^ Center for Infectious Diseases and Vaccine Research, West China Hospital, West China School of Medicine, Sichuan University, Chengdu, China; ^2^ Department of Healthcare Intelligence, University of North America, Fairfax, VA, United States

**Keywords:** *Mycobacterium tuberculosis*, therapeutic vaccine, antibody-dependent phagocytosis, Ag85B, Rv2660c, MPT70, polyfunctional T cells

## Abstract

Latent tuberculosis infection (LTBI), affecting nearly one-quarter of the global population, represents a major barrier to Tuberculosis (TB) eradication and a paradigm of chronic infectious disease. Current chemotherapeutic regimens for TB, although effective, are limited by drug resistance, toxicity, and poor adherence, underscoring the urgent need for alternative strategies. In this study, we investigated ARM—a recombinant fusion protein comprising Ag85B, Rv2660c, and MPT70—as a therapeutic vaccine in a murine model of post-exposure *Mycobacterium tuberculosis* (*Mtb*) infection. ARM immunization elicited robust CD4+ T cell responses, with a higher frequency of polyfunctional T cells producing IFN-γ, and TNF-α compared to the classical BCG vaccine. Critically, ARM also induced strong humoral immunity, marked by elevated *Mtb*- and ARM-specific IgG levels that enhanced FcγR-dependent phagocytosis, phagosome–lysosome fusion, and intracellular bacterial clearance. ARM-treated mice exhibited reduced pulmonary pathology, improved weight recovery, and superior control of bacterial burden. These findings demonstrate the potential of therapeutic vaccination to mobilize both cellular and antibody-mediated immunity in controlling *Mtb* infection and offer a broader immunological strategy for managing chronic infectious diseases. ARM represents a promising candidate for post-exposure TB vaccination, with potential to enhance bacterial clearance and reduce disease progression in high-burden populations.

## 1 Introduction

Tuberculosis (TB), cause by *Mycobacterium tuberculosis* (*Mtb*) infection, remains the leading cause of death from a single infectious agent, affecting over 10.8 million people annually with 1.25 million deaths in 2023 (1). Latent *Mtb* infection (LTBI) presents a major challenge to the elimination of the TB epidemic. A quarter of the global population is latently infected (2) and 10% of those progress to active TB annually, serving as a reservoir for active TB and a driver of ongoing TB transmission (3). While anti-TB chemotherapy is the only recommended countermeasure against LTBI, issues such as antibiotic resistance resistant (4), adverse effects (5, 6), and patient compliance (7) limit its effectiveness. Thus, alternative approaches to overcome these limitations or complement the classical treatment are urgently needed.

Therapeutic vaccination holds the potential to serve as adjunct to chemotherapy against TB (8). Post exposure administration of the vaccine could improve chemotherapy outcomes by enhancing immune-mediated control of *Mtb* replication and even eradicating the bacteria, provide opportunity to shorten and simplify the treatment and to prevent disease recurrence (9, 10). This immune-mediated control is supported by observation that 90% of infection do not progress to active disease progression (11, 12), and LTBI keeps the bacteria in control without progression in most cases (13). Although the majority of individuals who develop active TB are not overtly immunocompromised, and the precise triggers of reactivation remain incompletely understood (14), adverse conditions such as co-infections or immune suppression may trigger *Mtb* reactivation, leading to the disease progression (15, 16). These observations demonstrate that the host’s instinct ability to control TB. The host control of *Mtb* is primarily achieved through the activation of CD4+ T helper and CD8+ cytotoxic T cells, which contribute to granuloma formation and bacterial suppression (17). Th1 cytokines, such as interferon-gamma (IFN-γ) and tumor necrosis factor-alpha (TNF-α), are critical for initiating immune responses and are widely used in preclinical models as indicators of vaccine-induced immunity (18, 19). Although their correlation with protection in human studies has been differed (20, 21), these cytokines remain valuable indicators of immune activation and play mechanistic roles in maintaining infection control (22). Additionally, *Mtb*-specific antibodies play a critical role in promoting *Mtb* phagocytosis and clearance via opsonization and FcγR activation (23, 24), which synergically enhances *Mtb* elimination. Although based largely on passive immunization studies, monoclonal antibodies against mycobacterial proteins (25–27) and polysaccharides (28, 29) have been shown to reduce bacterial loads and pathology in mice. Synergistic effects with IFN-γ further support a potential protective role for antibodies (30). While not definitive for natural immunity, these findings highlight humoral responses as a promising component of TB protection. Given the suppressive effects of Th2 cytokines such as IL-4 on B cell function (31, 32), further studies are needed to determine how antibody functions can be effectively elicited and integrated into TB vaccine strategies.

Animal models have demonstrated that the effectiveness of therapeutic vaccination is associated with elevated CD4+ T cell activation, promoted effector cytokine release, enhanced natural killer cell cytotoxicity, and reduced myeloid-derived suppressor cells (33–35). Clinical trial of therapeutic vaccine candidates, including H56:IC31 (33, 36, 37), ID93+GLA-SE (38), and M72/AS01E (39), also demonstrated that robust and sustainable polyfunctional CD4+ T cell responses were associated with the potency of the therapeutic effect (40). Post-exposure vaccine has the potential to control epidemics by reducing latent infection, which in turn can reduce *Mtb* transmission and incidence. Mathematical modeling suggested that vaccination of *Mtb* exposed adults were more effective on reducing TB epidemic than vaccinating naïve newborns (41, 42), and could prevent more than 37 million TB incidences and 4–6 million deaths in the next decade (43). Thus, World Health Organization has prioritized post exposure vaccination as a primary strategy of TB epidemic control (44). The concept of post exposure vaccination has been proven successful in control of many infections, such as hepatitis A, hepatitis B, measles, and rabies and has being explored in diseases like anthrax, meningococcal meningitis, tetanus, pneumococcal disease and diphtheria (45), providing a solid theoretical foundation for post exposure vaccination in TB. However, effective therapeutic vaccines targeting *Mtb*-exposed populations remain elusive.

Although Bacillus Calmette-Guerin (BCG) has been administered to children to prevent severe forms of TB—primarily rare disseminated and meningeal disease in early childhood—it offers little protection against pulmonary TB and is not recommended for post exposure vaccination due to potential adverse reactions (46, 47). Most of the recombinant protein vaccine candidates focus on T cell immunity and have yet to address potency of immune effectors such as antibody-dependent cellular phagocytosis (ADCP), which could further enhance therapeutic efficacy (48), and there is an increasing acknowledgement of stimulating CD4+ T cell response alongside humoral immunity to improve effectiveness of therapeutic vaccination (24). In our previous report, we have developed a recombinant fusion protein that is composed of *Mtb* components Rv2660c, Ag85B, and MPT70 (ARM). Ag85B is a well-characterized secreted vaccine antigen that can induces both humoral and CD4+ T cell responses (36, 37); Rv2660c is a cytosolic protein upregulated under starvation conditions and is associated with *Mtb* dormancy, and can stimulates both CD4+ and CD8+ T cell responses (33, 49–52); MPT70 is a membrane-associated protein that can triggers CD4+ T cell and B cell responses (53–55), while also contributing to cytotoxic T cell activation (55, 56). These immunodominant antigens elicited robust *Mtb*-specific CD4 T cell and B cell responses, and facilitated *Mtb* clearance (57), which makes ARM a promising candidates for therapeutic vaccine.

In this study, we assessed ARM boosted *Mtb*-specific immune responses in the H37Ra exposed mice, in terms of CD4+ T cell activation, multifunctional Th1 cell differentiation, cytokine releases, antibody production, and antibody-dependent *Mtb* phagocytosis, and compared to those induced by BCG. We also evaluated the role of the immune responses on *Mtb* clearance and infection-induced inflammatory pathology. Although H37Ra lacks several virulence determinants of H37Rv, it has been widely used for the preliminary evaluation of anti-TB drugs (58, 59) and experimental TB vaccine candidates (60, 61). Therefore, our results may shed a light on the application of ARM as a promising therapeutic vaccine for *Mtb*-exposed populations and underscore the importance of boosting both T and B cell-mediated immunity in TB vaccine design and development.

## 2 Materials and methods

### 2.1 *Mycobacterium* strains and mice

Bacille Calmette-Guerin (BCG) strain Pasteur 1173P2 and *M. tuberculosis* strain H37Ra were purchased from Shanghai Gene-Optimal Science and Technology (Gene-Optimal, China), and cultured on Middlebrook 7H11 agar plate or in 7H9 medium (BD Biosciences, USA) supplemented with 10% *BBL* Middlebrook *OADC* enrichment *(*BD Biosciences, USA*)*. Six- to eight-week-old female specific-pathogen-free (SPF) C57BL/6 mice were purchased from the Experimental Animal Center of Sichuan University (Sichuan University, China) and housed under pathogen-free conditions with independent ventilation at West China Hospital, Sichuan University, China. The protocol of animal experiment was reviewed and approved by the Institutional Animal Care and Use Committee (IACUC), West China Hospital of Sichuan University (No. 20220228076).

### 2.2 Recombinant ARM cloning and expression

The *ag85B-rv2660c-mpt70 (arm)* DNA segment was amplified from previously constructed pGAPZαA-*arm* recombinant plasmids (57) using specific primers (5’-CCGGAATTCCGTCCAGGTTTGCCAG-3’ and 5’-CCGCTCGAGAGCTGGTGGCATCAAAA-3’) that ligating to the incorporated EcoRI and XhoI restriction enzyme sites. The amplification employed PrimeSTAR Max DNA Polymerase (Takara, Japan) and followed the manufacture’s instruction. The amplified product was purified with a TIANgel Purification Kit (TIANGEN, China) and inserted into bacterial expression vector pET-28a (+) using non-ligase-dependent ClonExpress II One Step Cloning Kit (Vazyme, China) after digestion of EcoRI and XhoI (Takara, Japan). Recombinant pET-28a (+)-*arm* plasmids were confirmed by Sanger sequencing (Tsingke, China) and transformed into *E. coli* BL21(DE3) cells (Tsingke, China) by heat shock. Protein expression was induced with 0.5 mM IPTG for 4 hours at 37°C, yielding recombinant proteins as inclusion bodies. Precipitate was washed and dissolved in 8 M urea buffer (Buffer A) before purification using Ni²+ affinity columns (Bio-Rad, USA). Purified proteins were dialyzed against storage buffer (25 mM NaH_2_PO_4_, pH 8.0, 10% glycerol, 150 mM NaCl, 0.05% Tween20), quantified, and analyzed for purity using SDS-PAGE and western blot.

### 2.3 Mouse infection, immunization and bacterial load assessment

C57BL/6 mice were infected with 1 × 10^7^ CFU of *M. tuberculosis* H37Ra in 0.1 mL phosphate buffered saline (PBS) via tail vein injection. Uninfected mice were inoculated with the same volume of PBS as a naive control. Four weeks later, the infected mice were randomly divided into three groups. The PBS group received 2 doses of 100 μL PBS with 30 μg CpG ODN 1826 (Invivogen, USA) intramuscularly at two-week intervals; The ARM group received 2 doses of 10 μg ARM protein in 100 μL PBS with 30 μg CpG ODN 1826 intramuscularly at two-week intervals; The BCG group received 1 dose of 1 × 10^6^ CFU BCG in 100 μL PBS subcutaneously along the midline on the back. The intramuscular injection site was at the posterior thigh blank muscle. The body weight was monitored weekly and reported as the percentage of initial weight before the infection. To assess bacterial loads, mice were sacrificed at indicated time intervals. The lung and spleen were removed and homogenized in 1 mL PBS using gentleMACS M tube (Miltenyi, Germany). The homogenized tissue suspension was serially 10-fold diluted and plated on 7H11 agar plates supplemented with 10% OADC. Colony-forming units (CFU) were counted after incubation at 37°C for three weeks and reported as log_10_ CFU.

### 2.4 Assessment of mononuclear cytokine expression by flow cytometry

Lung and spleen were collected from mice and processed into single-cell suspensions using GentleMACS dissociators and 70-μm cell strainer (Miltenyi, Germany). The mononuclear cells were isolated using Ficoll (Cytiva, USA) gradient centrifugation. After washing, cells were seeded into U-bottom 96-well plate (Thermofisher, USA) at 2×10^6^ cells/well and cultured with 20 μg/mL ARM or 25 μg/mL H37Rv whole-cell lysate (WCL, Gene-Optimal, China) in the presence of 1 μg/mL anti-CD28/CD49d (eBioscience, USA) at 37°C in 5% CO_2_ for 3 hours. The culture was added with GolgiStop (eBioscience, USA) at final concentration of 1 μg/mL and incubated for another 11 hours. Cells were harvested and washed twice in cold Dulbecco’s modified phosphate-buffered saline (DPBS), then stained with surface marker antibodies diluted in 50% brilliant stain buffer (BD Horizon, USA) and fixable viability stain 700 (BD Biosciences, USA) (1:1000) at 4 °C for 20 min. After washing, cells were fixed and permeabilized with the Cytofix/Cytoperm Fixation/Permeabilization Kit (BD Biosciences, USA) according to manufacturer’s instructions, followed by intracellular cytokine staining for IFN-γ, TNF-α, IL-2, and IL-17A at 4 °C for 30 min. Data were collected using a FACSymphon A5 (BD Biosciences, USA) and analyzed using FlowJo v10.6.2 (BD FlowJo, USA). The following fluorochrome-conjugated antibodies were used for flow cytometric assessment: CD45-FITC (BioLegend, clone: 30-F11), CD3-APC/Cy7 (BD Biosciences, clone: 145-2C11), CD4-BV605 (BD Biosciences, clone: RM4-5), CD8-PerCP/Cy5.5 (BD Biosciences, clone: RPA-T8), CD44-BUV395 (BD Biosciences, clone: IM7), CD62L-APC (BioLegend, clone: MEL-14), CD69-BV510 (BioLegend, clone: H1.2F3), CD103-BV421 (BioLegend, clone: 2E7), IFN-γ-PE (BioLegend, clone: XMG1.2), TNF-α-PE/Cy7 (BioLegend, clone: MP6-XT22), IL-2-BV711 (BioLegend, clone: JES6-5H4), and IL-17A-BV785 (BioLegend, clone: TC11-18H10.1).

### 2.5 Enzyme-linked immunosorbent assay of serum antibody and cytokine

For antibody assay, polystyrene 96-well plates (Corning, USA) were coated with 1 μg/mL ARM or WCL protein in 100 μL Carbonate-Bicarbonate buffer (pH 9.6) overnight at 4°C. The plates were then washed three times with PBS containing 0.1% Tween-20 (PBST) and blocked with 3% BSA in PBST at 37°C for 2 hours. A volume of 100 μL serum sample in 5-fold serial dilutions, starting from 1:50, was added to the plate followed by incubation at 37°C for 1 hour. After washing, the plates were incubated with 1:5000 diluted HRP-conjugated antibodies (Proteintech, China) in 100 μL PBST for 1 hour and developed with 100 μL TMB substrate (Solarbio, China) for 5 minutes. The reaction was stopped with 100 μL 0.5 M H2SO4. The optical density (OD) was measured at 450 nm using a SpectraMax i3x microplate reader (Molecular Devices, USA). Endpoint titers were defined as the highest dilution with OD ≥ 2× negative control. For cytokine assay, serum TNF-α, IFN-γ, IL-2, and IL-10 were quantified using mouse cytokine ELISA kits (Ruixin, China) following the manufacture’s instruction.

### 2.6 Construction and cloning of recombinant H37Ra-eGFP strain

H37Ra cells at density of OD_600_ 0.5-1.0 in 1 mL were transferred to 100 mL 7H9 culture medium supplemented with 10% OADC (BD) and cultured for 2 weeks at 37°C. After centrifugation at 3000×g for 10 minutes, the pellet was resuspended in 10% glycerol and washed twice by centrifugation. Finally, the pellet was resuspended in 2 mL of 10% glycerol to prepare H37Ra competent cells. The H37Ra competent cells in 100 μL were loaded into a 2 mm Gene Pulser cuvette (Bio-Rad, USA) and mixed with 10 μL of pMV-261-eGFP plasmid (Gene-Optimal, China). Transformation was performed using the Gene Pulser Xcell™ (Bio-Rad, USA) at 2.5 kV/cm, 25 µF, 1000 Ω. Colonies were grown on 7H11 agar plates (BD Biosciences, USA) supplemented with 10% OADC enrichment, 0.5% glycerol, and 50 µg/mL kanamycin at 37°C for 35 days. Positive colonies expressing eGFP were verified by fluorescence microscopy and Ziehl-Neelsen acid-fast staining.

### 2.7 Macrophage phagocytosis and inhibition assay

Murine macrophage Raw264.7 cells were suspended in DMEM medium (Gibco, USA) supplemented with 10% heat-inactivated fetal bovine serum (FBS, Gibco, USA), seeded into 96-well tissue culture plates at density of 1×10^5^ cells/well, and incubated at room temperature for 3 hours. The plate was gently washed with the culture medium to remove untouched cells and incubated for another 2 hours at 37°C in DMEM medium supplemented with 10% heat-inactivated mouse serum samples in duplicate wells. H37Ra-eGFP was added at a multiplicity of infection of 20 into each well and incubated for 3 hours. The plate was washed with PBS and dissociated the monolayer cell with 0.04% EDTA-PBS. The Raw264.7 cells with phagocytosed H37Ra-eGFP were detected with by flow cytometry.

For assessing inhibitory effect of macrophages on mycobacteria, Raw264.7 cells were cultured with H37Ra with a MOI of 10 in the presence of mouse serum samples as described above and incubated for 3 hours. After washing, the Raw264.7 cells were treated with 100 µg/mL gentamycin for 1 hour to eliminate extracellular bacteria. For cells harvested on day 1, 0.05% SDS was added to lyse the cells, and the lysates were serially 10-fold diluted and plated on Middlebrook 7H11 agar plates. For cells harvested on day 4, 100 µL of DMEM medium containing 10% mouse serum was added to each well after un-engulfed bacteria were removed on day 1. Supernatants were pooled, and the cells were lysed and plated on 7H11 agar plates on day 4. Colony-forming units (CFUs) were counted after incubating the plates at 37°C for 3 weeks, with a second count performed at 4 weeks to check for additional visible colonies.

### 2.8 Histological examination

Lung tissues were fixed in 4% paraformaldehyde, embedded in paraffin and sectioned. The section samples were stained with hematoxylin and eosin and examined under microscope (Olympus, Japan). Histopathological changes in lung were evaluated by blinded investigators using a scoring system (62) based on inflammatory cell infiltration (scale 0-3), alveolar structure destruction (scale 0-3), hemorrhage or vascular congestion (scale 0-2), and fibrosis (scale 0-2). The sum of respective scales with a total of maximum 10 represents severity of pathology.

### 2.9 Statistical analyses

GraphPad Prism version 9.0 was used for statistical analysis and graphical presentation of data. Data were displayed as mean ± SD. One-way and two-way ANOVA were used for group and subgroup analysis respectively. P values ≤ 0.05 were considered significant.

## 3 Results

### 3.1 Post-exposure administration of ARM facilitates H37Ra clearance

Our previous study reported the construction of a nucleic acid segment encoding the recombinant *Mycobacterium tuberculosis* Ag85B, Rv2660c, and MPT70 fusion protein (ARM) and its immunogenicity in mice (57). In this study, we constructed a pET-28a (+)-*arm* expression vector and expressed the recombinant ARM protein using *E. coli* BL21 (DE3). SDS-PAGE and western blot analyses confirmed the recombinant product as a 59 kDa fragment with specific 6×His-tag binding, consistent with predicted ARM characteristics, and the purity exceeding 94.5% (**Supplementary Figure S1A**). To establish a mouse model of *Mtb* infection, female SPF mice (6–8 weeks old) were infected with 10^6^, 10^7^, or 10^8^ CFU of H37Ra via tail vein injection. Weight changes were monitored weekly, and bacterial loads in the lungs and spleen were assessed on days 1, 7, 28, 42, and 84 post-infection. After infection, mice exhibited dose-dependent weight loss that persisted for at least 84 days without recovery compared to controls (**Supplementary Figure S1B**). Bacterial loads in the lungs and spleen peaked on day 42 and subsequently declined, correlating positively with the inoculation dose (**Supplementary Figure S1C**). Based on weight loss severity and pathogen persistence, 10^7^ CFU was selected as the optimal dose for the mouse model.

**Figure 1:**
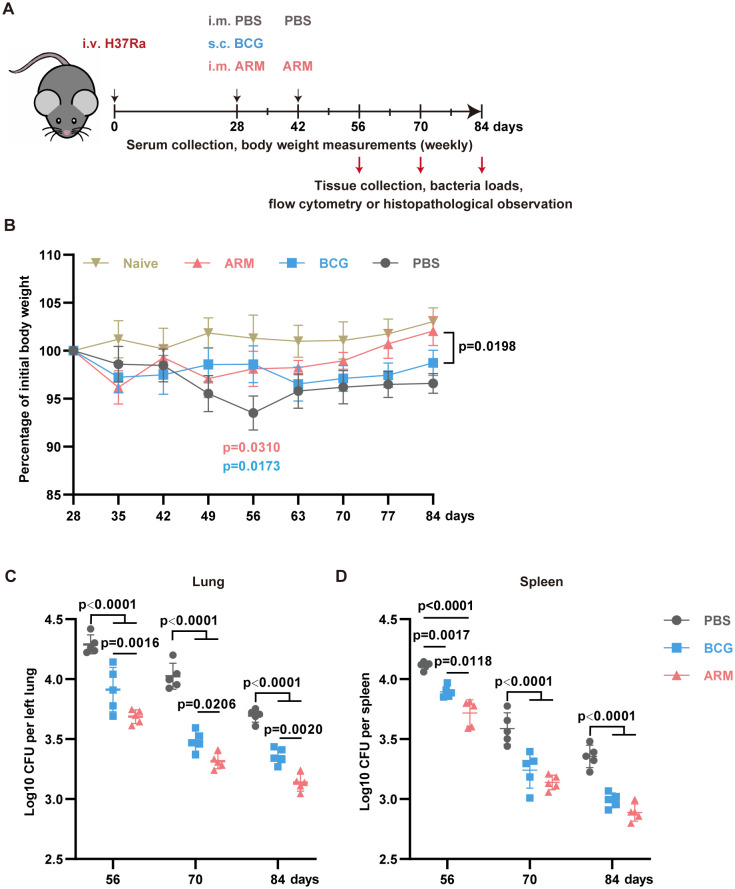
Therapeutic administration of ARM-fusion protein enhances *Mtb* clearance in pre-exposed mice. **(A)** Schematic overview of the experimental timeline. Mice were infected with *Mycobacterium tuberculosis* and treated at 4 weeks post-infection with either PBS, ARM (10 μg), or BCG (10^6^ CFU) via the indicated routes. Bacterial burden and immune responses were evaluated on days 56, 70, and 84. **(B)** Body weight changes over time, expressed as a percentage of the initial body weight at the start of immunization. Treatments were administered on day 28, with a booster dose of PBS or ARM on day 42. Weight was monitored weekly until day 84 (n = 5). **(C)** Lungs (left) and spleen (right) bacterial loads, presented as Log_10_ CFU, at the indicated time points (n = 5). Data are presented as mean, with error bars representing the standard deviation (SD) and analyzed by one-way or two-way ANOVA with multiple comparisons. P values are denoted in the figure.

In this model, on day 28 post-infection (exposure), mice were treated with either PBS+30 μg CpG, 10 μg ARM+ 30 μg CpG, or 10^6^ CFU BCG, all in 100 μL PBS as described in the methods (**Figure 1A**). Mice treated with ARM and BCG stopped losing weight by day 35, one week after treatment. In contrast, PBS-treated mice continued to lose weight until day 56 post-infection. ARM-treated mice regained weight comparable to uninfected control, while BCG-treated mice maintained a lower but stable weight compared to both the uninfected control and ARM-treated mice (**Figure 1B**). Regarding mycobacterial load, both ARM- and BCG-treated mice showed significantly lower H37Ra loads in the lungs and spleen than PBS-treated mice. ARM-treated mice exhibited significantly lower H37Ra loads in the lungs on all tested days and in the spleen on day 56 compared to BCG-treated mice. Additionally, ARM-treated mice demonstrated lower H37Ra loads in the spleen on days 70 and 84, although these reductions were not statistically significant compared to BCG group (**Figure 1C**).

### 3.2 Post-exposure administration of ARM mitigates H37Ra-induced pulmonary pathology

**Figure 2:**
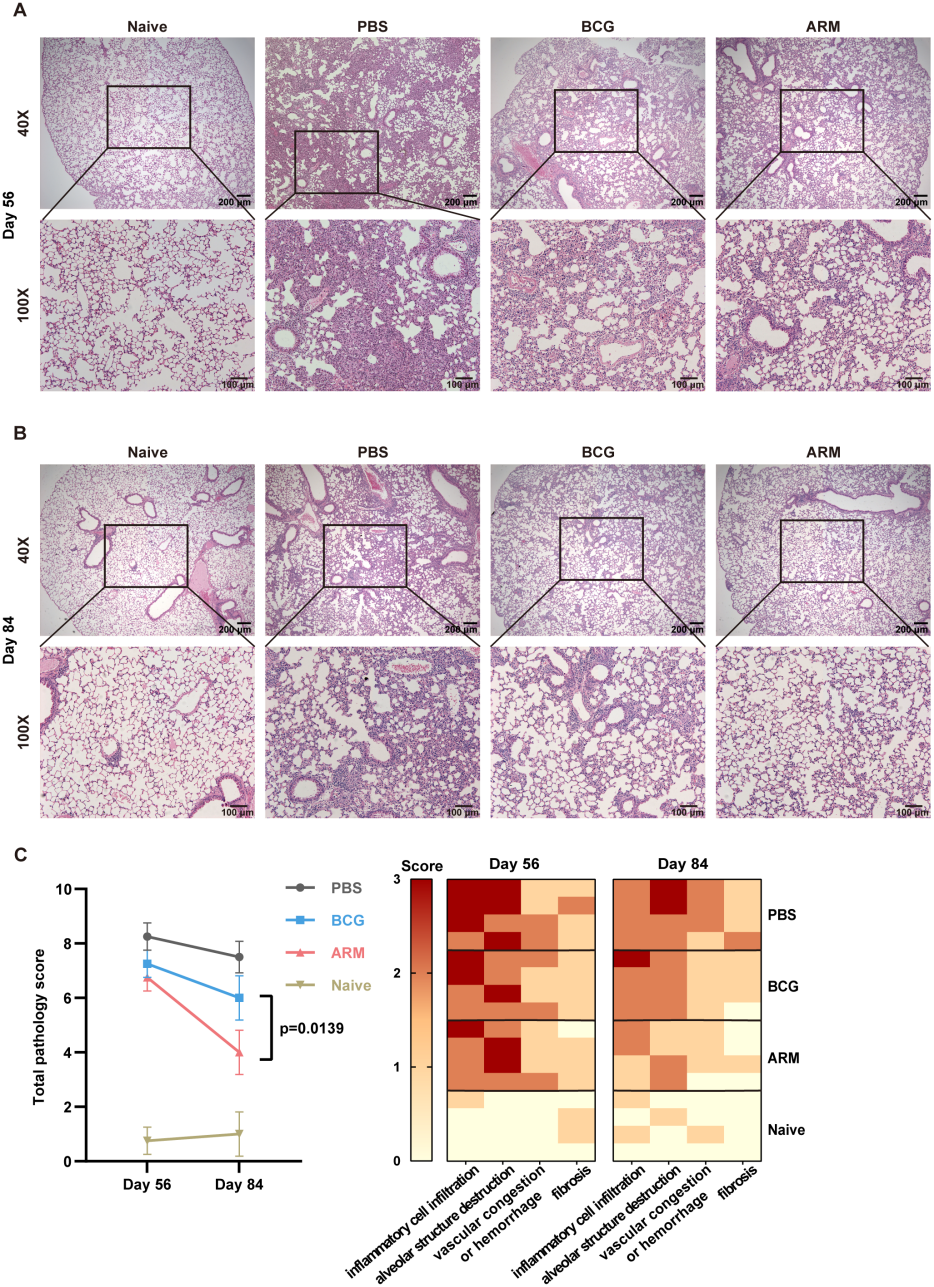
ARM-fusion protein mitigates pulmonary pathology in *Mtb*-exposed mice. **(A, B)** Representative H&E-stained lung sections from uninfected controls (Naive) and *Mtb*-exposed mice treated with PBS, ARM, or BCG at days 56 **(A)** and 84 **(B)** post-infection. **(C)** Histological scores of lung tissue sections at days 56 and 84 post-infection. The total pathology score was calculated as the sum of four parameters: inflammatory cell infiltration, alveolar structure destruction, hemorrhage or vascular congestion, and fibrosis, with a maximum score of 10. Higher scores indicate more severe pathological changes. (n = 4). Data are presented as mean, with error bars representing the standard deviation (SD) and analyzed using one-way ANOVA with multiple comparisons. P values are indicated.

On day 56 post-infection, lung histological analysis of PBS-treated mice revealed widespread alveolar collapse, mononuclear cell infiltration in the parenchyma, thickened alveolar septa, inflammatory cell aggregates adjacent to bronchioles and vasculature, and patchy hemorrhage in the alveolar space. In contrast, post-infection treatment with ARM and BCG markedly alleviated pathological inflammation, as indicated by reduced alveolar destruction, decreased inflammatory infiltration, thinner alveolar septa, and the absence of hemorrhage. Among these groups, ARM-treated mice exhibited less severe pathological inflammation than the BCG group (**Figure 2A**). These differences became more pronounced on day 84 relative to day 56 (**Figure 2B**). Pathological severity was quantitatively evaluated by blinded investigators using an arbitrary scoring system (**Figure 2C**).

### 3.3 ARM enhances cytokine lung and spleen T cell responses in *Mtb*-exposed mice

T cell responses, particularly CD4+ T cell response, are crucial for controlling *M. tuberculosis* infection (16). To investigate the impact of ARM on *Mtb*-specific T cell responses, mononuclear cells were isolated from the lungs and spleen on day 28 post-immunization (day 56 post-infection) and stimulated *in vitro* with H37Rv whole-cell lysate proteins. Cytokine expression was detected using intracellular cytokine staining and quantified by flow cytometry. Activated CD44+ T cells were gated and the cytokine production was assessed as shown in **Supplementary Figure S2A**. The frequency of CD44+ cells among total CD4+ T cells was significantly higher in ARM-treated mice compared to both PBS and BCG groups. While the proportion of CD44+ cells within the CD8+ T cell population was comparable to that in the BCG group but remained significantly higher than in the PBS group. (**Supplementary Figure S2B**).

**Figure 3:**
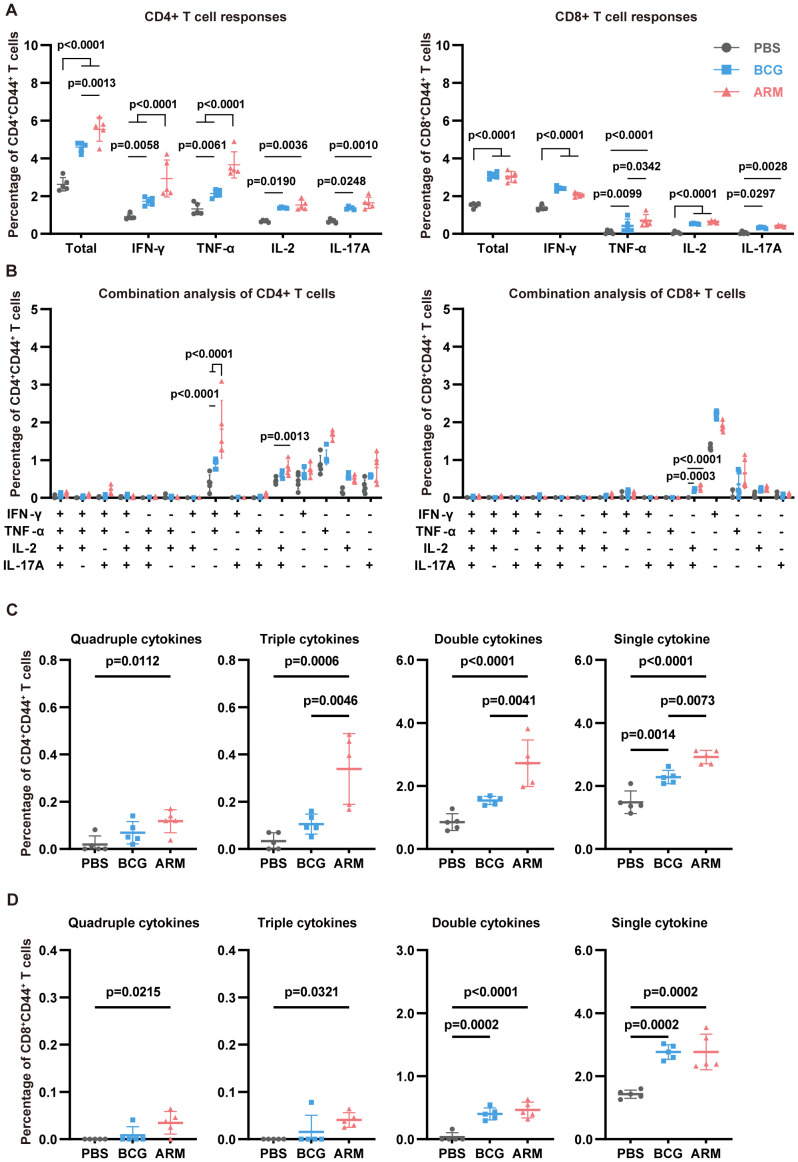
ARM-fusion protein enhances lung T cell responses in *Mtb*-exposed mice. **(A)** Frequencies of cytokine-producing CD44+CD4+ and CD44+CD8+ T cells in the lungs at day 56 post-infection. Mononuclear cells were isolated and stimulated with H37Rv whole-cell lysate (WCL). Cytokine expression was assessed using intracellular staining with fluorescence-conjugated antibodies and analyzed by flow cytometry (n = 5). **(B)** Combination analysis of multiple cytokine-producing CD44+CD4+ and CD44+CD8+ T cells in the lungs (n = 5). **(C, D)** Proportions of T cells producing four, three, two, or one cytokine. (n = 5). The gating strategy is shown in **Supplementary Figure S2**. Data are presented as mean, with error bars representing the standard deviation (SD) and analyzed using two-way ANOVA with multiple comparisons. P values are denoted in the figure.

Upon stimulation with H37Rv whole-cell lysate (WCL), lung CD4+CD44+ T cells from both BCG- and ARM-treated mice demonstrated significantly elevated frequencies of IFN-γ-, TNF-α-, IL-2-, and IL-17A-producing cells compared to the PBS group (**Figure 3A**, left). Notably, ARM vaccination induced a significantly higher proportion of IFN-γ+ and TNF-α+ CD4+ T cells compared to BCG, suggesting a stronger Th1-type immune response. In terms of polyfunctional T cell responses, ARM-treated mice exhibited markedly increased frequencies of multi-cytokine-producing CD4+ T cells, especially those simultaneously expressing two or three cytokines, while BCG treatment mainly promoted the generation of single-cytokine-producing T cells (**Figure 3C**). The most common combination among CD4+ T cells was IFN-γ+TNF-α+, indicating coordinated cytokine responses (**Figure 3B**, left). For CD8+CD44+ T cells (**Figure 3A**, right), both BCG and ARM immunizations significantly enhanced the production of IFN-γ, TNF-α, IL-2, and IL-17A, with ARM again inducing a higher proportion of TNF-α+ CD8+ T cells than BCG. Consistent with CD4+ T cell data, ARM treatment also led to increased frequencies of polyfunctional CD8+ T cells, particularly double cytokine combinations, compared to PBS (**Figure 3D**). The most frequent combination in this subset was IL-2+IL-17A+ (**Figure 3B**, right), highlighting a distinct cytokine co-expression pattern that may contribute to enhanced pulmonary immunity.

**Figure 4:**
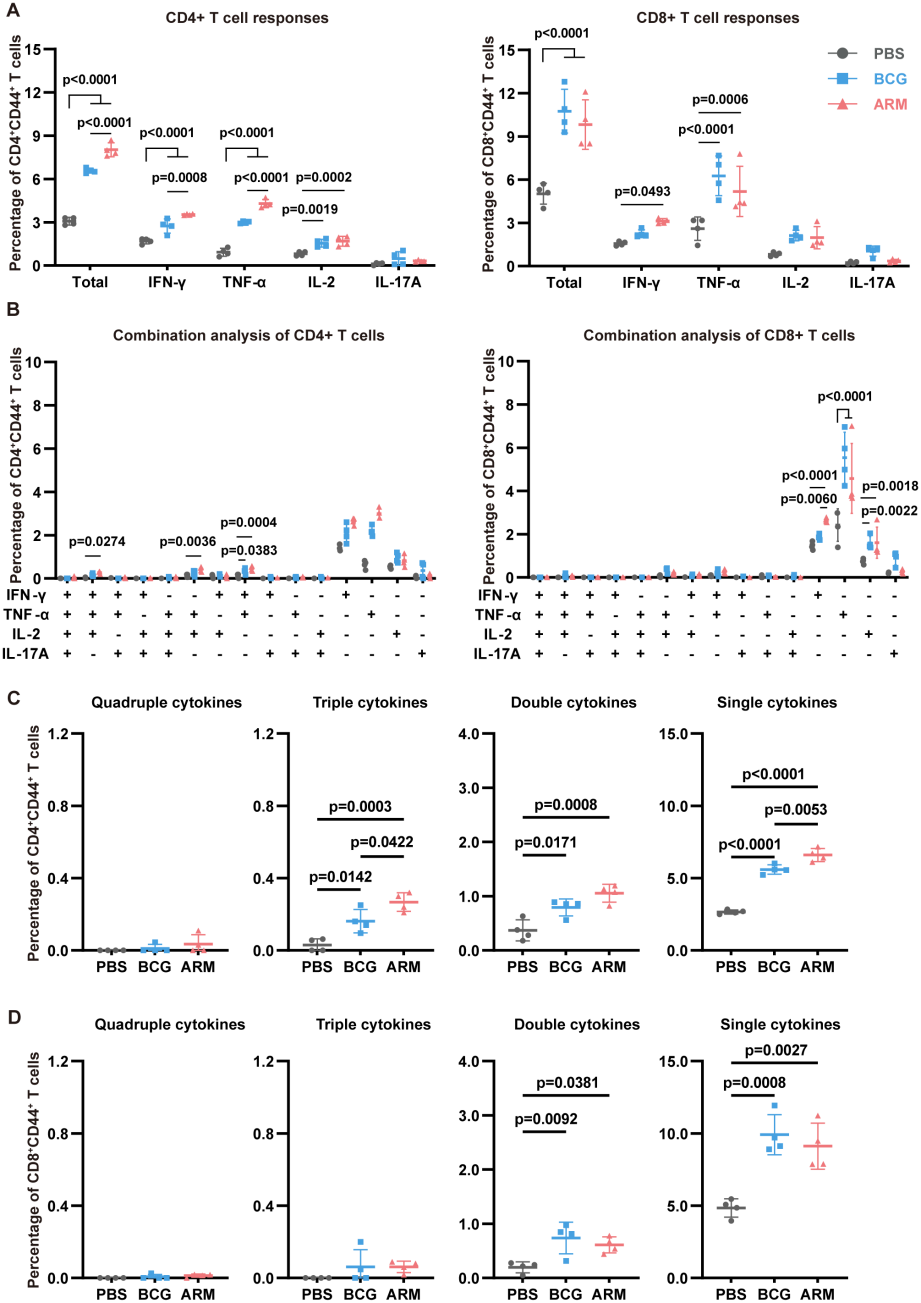
ARM-fusion protein promotes spleen T cell responses in *Mtb*-exposed mice. **(A)** Frequencies of cytokine-expressing CD44+CD4+ and CD44+CD8+ T cells in the spleen at day 56 post-infection after H37Rv-WCL stimulation (n = 4). **(B)** Polyfunctionality analysis of CD4+ and CD8+ T cells (n = 4). **(C, D)** Distribution of T cells producing four, three, two, or one cytokine (n = 4). Data are presented as mean, with error bars representing the standard deviation (SD). Statistical significance was assessed using one-way or two-way ANOVA. P values are indicated.

Likewise, spleen CD4+CD44+ T cells from both BCG- and ARM-treated mice exhibited significantly elevated frequencies of IFN-γ+, TNF-α+, and IL-2+ cells compared to the PBS group (**Figure 4A**, left), with ARM induced a significantly higher proportion of IFN-γ+ and TNF-α+ CD4+ T cells than BCG, again indicating of a stronger Th1-type immune response. Further analysis of polyfunctional CD4+ T cell subsets (**Figures 4C**) demonstrated that both ARM and BCG increased the frequency of triple and double cytokine-producing cells compared to PBS, with ARM eliciting the highest levels of triple cytokine CD4+ T cells. Among these, the most prominent combinations included IFN-γ+TNF-α+IL-2+, IFN-γ+TNF-α+, and TNF-α+IL-2+ (**Figure 4B**, left), underscoring coordinated Th1 cytokine co-expression. In the CD8+CD44+ T cell population (**Figure 4A**, right), both BCG and ARM immunization significantly increased the overall frequency of cytokine-producing cells compared to PBS, although IL-2+ and IL-17A+ subsets remained relatively low and comparable among all groups. In line with CD4+ T cell trends, ARM and BCG also increased the frequency of polyfunctional CD8+ T cells, especially double cytokine producers (**Figure 4D**). While single cytokine-producing cells predominated across all groups, their frequencies were markedly elevated in both vaccinated groups, with the highest levels observed in ARM-treated mice for CD4+ T cells. These findings collectively suggest that ARM immunization enhances both the magnitude and functional quality of systemic T cell responses.

### 3.4 ARM elevates effector cytokines level in serum

**Figure 5:**
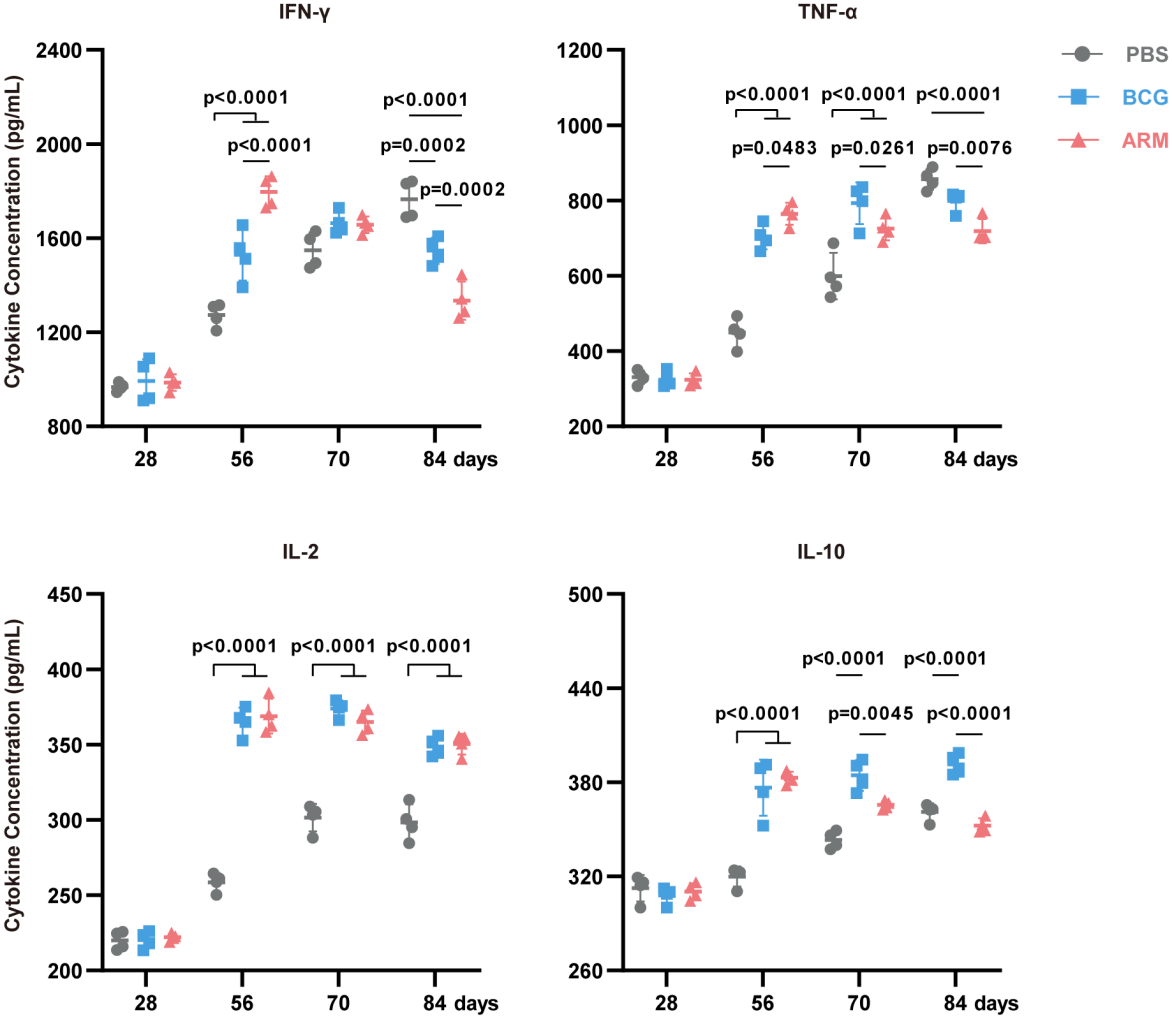
ARM-fusion protein elevates effector cytokines level in serum. Serum cytokine concentrations measured by ELISA at days 28, 56, 70, and 84 post-infection. (n = 4 mice per group). Data are presented as mean, with error bars representing the standard deviation (SD) and analyzed using two-way ANOVA with multiple comparisons. P values are shown.

To evaluate the systemic cytokine milieu following immunization, serum levels of key Th1 cytokines (IFN-γ, TNF-α, IL-2) and the regulatory cytokine IL-10 were measured over time using ELISA (**Figure 5**). Both BCG and ARM treatments significantly increased the concentrations of IFN-γ, TNF-α, and IL-2 compared to the PBS control across all time points. Notably, ARM treatment led to an earlier peak in IFN-γ levels at day 56, which was significantly higher than both the PBS and BCG groups. In contrast, the BCG-induced IFN-γ response peaked later, at day 70, but remained elevated through day 84. For IL-2, both BCG and ARM groups exhibited significantly elevated serum levels from day 56 onward, maintaining high concentrations through day 84. The PBS group displayed only a modest increase in IL-2 and remained significantly lower than both vaccine groups throughout the study period. Regarding IL-10, both BCG and ARM immunizations triggered a marked increase by day 56 compared to PBS. However, while BCG maintained high IL-10 levels over time, ARM-treated mice demonstrated a gradual decline in IL-10 concentrations post-day 56. This decline suggests that ARM may promote the resolution of inflammation more efficiently than BCG. Importantly, the temporal kinetics of serum Th1 cytokine production closely mirrored the dynamics of antigen-specific T cell responses observed in the lungs and spleens, particularly at day 56. The coordinated rise in serum cytokines and the frequency of cytokine-producing T cells suggests that ARM immunization not only enhances cellular immune responses but also modulates systemic cytokine profiles, potentially contributing to a balanced and effective protective immunity.

### 3.5 ARM elevates antigen-specific antibody level and enhances macrophage opsonized phagocytosis

**Figure 6:**
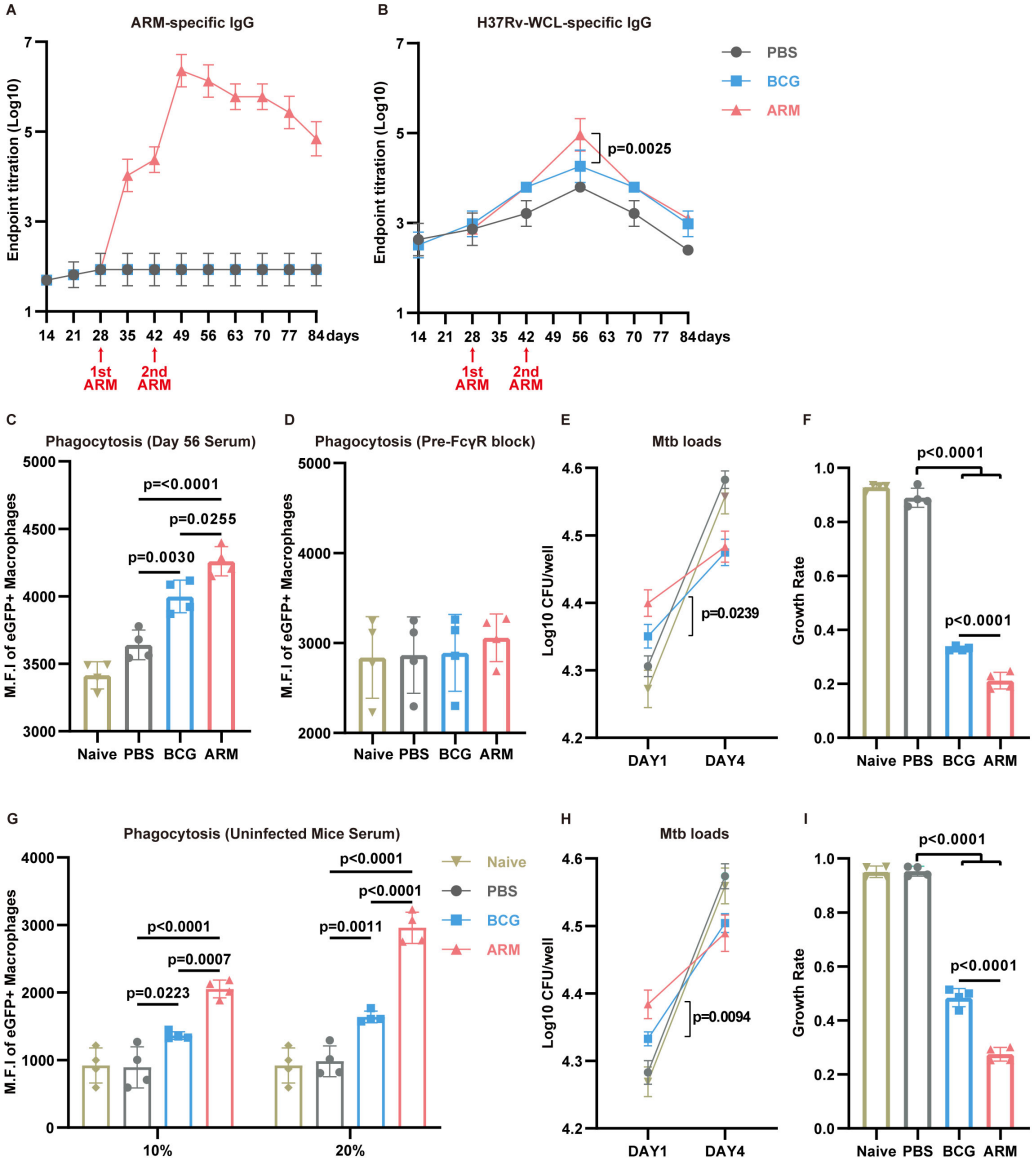
ARM-fusion protein elicits humoral immune responses to facilitate *Mtb* clearance. **(A)** Serum titers of ARM-specific IgG measured by ELISA (n = 6). **(B)** Serum titers of *H37Rv*-WCL-specific IgG (n = 6). **(C)** Phagocytosis assay using *H37Ra*-eGFP and Raw264.7 cells incubated with serum from Naive or *Mtb*-exposed mice treated with PBS, BCG, or ARM (n = 4). **(D)** Phagocytosis assay with pre-blocking of FcγRs. Cells were pre-incubated with anti-CD16/32 antibodies for 30 minutes before exposure to mouse serum (n = 4). **(E, F)** *Mtb* growth inhibition assay. CFUs at day 1 and day 4 were presented as Log10 values, and growth rates were calculated as CFU (day 4 − day 1)/day 1. Lower growth rates indicate greater growth inhibition (n = 4). **(G)** H37Ra-eGFP phagocytosis assay using Raw264.7 cells. Cells were incubated with serum from healthy controls (Naive) or non-*Mtb*-exposed mice treated with PBS, BCG, or ARM at day 28, at the indicated serum concentrations (n = 4). **(H, I)** *Mtb* growth inhibition assay. CFUs at day 1 and day 4 were presented as Log10 values, and growth rates were calculated as CFU (day 4 − day 1)/day 1. Lower growth rates indicate stronger growth inhibition (n = 4). Data are presented as mean, with error bars representing the standard deviation (SD) and analyzed using one-way ANOVA with multiple comparisons. P values are indicated in the figure.

Although the protective role of humoral immunity against intracellular pathogens remains under debate (23, 24), accumulating evidence supports antibody-mediated immunity against *Mtb* (25, 63–65). To investigate this, we assessed the humoral immune responses in *Mtb*-exposed mice subjected to different immunizations (**Figure 6**). ARM immunization led to a robust increase ARM-specific IgG titers, with a sharp rise following the first and second doses and peaking around day 49 (**Figure 6A**). ARM immunization markedly increased IgG (predominantly IgG2c) and IgM levels (**Supplementary Figure S3A**). Endpoint titers revealed that Ag85B-specific IgG was the most abundant (**Supplementary Figure S3B**). In contrast, neither PBS nor BCG induced detectable ARM-specific antibodies.

To evaluate the broader antibody response against *Mtb*, H37Rv-WCL-specific IgG levels were measured (**Figure 6B**). Although WCL-specific IgG levels peaked in all groups at day 56, the ARM group exhibited significantly higher titers compared to BCG, indicating an enhanced humoral response. To determine the functional capacity of these antibodies, we performed antibody-dependent phagocytosis assays using day 56 sera. RAW264.7 cells incubated with sera from ARM-treated mice exhibited a substantial increase in mean fluorescence intensity (MFI) of H37Ra-eGFP (**Figure 6C**), indicating enhanced phagocytosis. This effect was markedly diminished upon Fcγ receptor (FcγR) blockade, highlighting the crucial role of the antibody-FcγR pathway in promoting *Mtb* phagocytosis (**Figure 6D**). We next assessed the impact of antibody responses on bacterial killing. Sera from ARM-treated mice led to significantly higher *Mtb* uptake at day 1, as shown by increased CFU counts (**Figure 6E**), consistent with enhanced phagocytosis. Growth rates, calculated as CFU (day 4-day 1)/day 1, showed that sera from ARM-treated mice significantly inhibited *Mtb* growth compared to BCG and PBS sera (**Figure 6F**).

Given the complex composition of serum from *Mtb*-exposed mice, we conducted additional functional assays using sera from non-*Mtb*-exposed mice immunized with PBS, BCG, or ARM at the same time points to further validate these findings. Phagocytosis assays revealed that both BCG and ARM sera enhanced *Mtb* uptake in a dose-dependent manner, with ARM showing superior activity (**Figure 6G**) CFU assays showed that ARM sera significantly restricted *Mtb* growth (**Figures 6H–I**), reinforcing the notion that ARM immunization generates functional antibodies capable of promoting phagocytosis and limiting intracellular bacterial replication.

### 3.6 Antibody opsonization facilitates phagosome-lysosome fusion in macrophages

**Figure 7:**
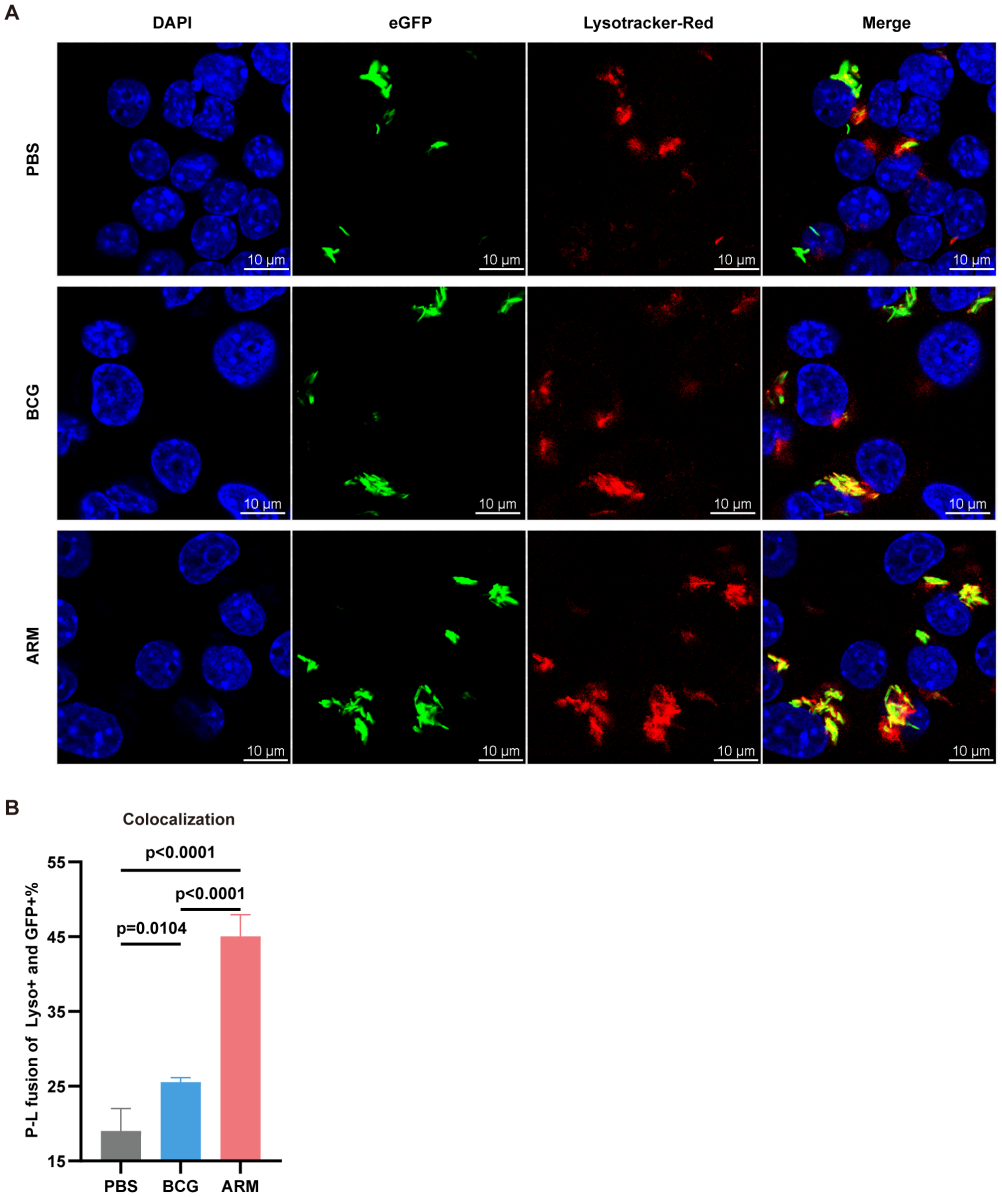
ARM-fusion protein promotes antibody-dependent phagocytosis and phagosome-lysosome fusion. **(A)** Representative confocal microscopy images from H37Ra-eGFP phagocytosis assays using Raw264.7 cells. Colocalization of H37Ra-eGFP (green) and LysoTracker (red) indicates phagosome-lysosome (P-L) fusion (yellow). **(B)** Quantification of P-L fusion, presented as the percentage of LysoTracker+eGFP+ phagosomes. A higher colocalization index suggests enhanced FcγR-mediated fusion due to increased ARM-specific antibody reactivity (n = 4). Data are presented as mean, with error bars representing the standard deviation (SD) and analyzed using one-way ANOVA with multiple comparisons. P values are indicated.

Phagocytosis followed by phagosome–lysosome (P–L) fusion is a critical step in macrophage-mediated elimination of *Mtb*, facilitating bacterial degradation within the phagolysosome (66). To further investigate the mechanisms underlying enhanced bacterial clearance, we assessed P–L fusion in RAW264.7 macrophages treated with sera from PBS-, BCG-, or ARM-immunized mice. Confocal microscopy revealed markedly increased colocalization of H37Ra-eGFP (green) with LysoTracker-labeled lysosomes (red) in the ARM group, as evidenced by the pronounced yellow signal in the merged images (**Figure 7A**). Quantitative analysis confirmed a significantly higher percentage of colocalization in the ARM group compared to both PBS and BCG controls (**Figure 7B**), indicating more efficient P–L fusion. These results suggest that antibodies induced by ARM immunization enhance both *Mtb* uptake and subsequent lysosomal fusion, contributing to improved intracellular bacterial clearance.

## 4 Discussion

In this study, we evaluated the effectiveness of ARM as a therapeutic vaccine in a *Mtb* post exposure mouse model and compared it with BCG, a classical TB vaccine. Both the ARM and the BCG could facilitate H37Ra clearance, and mice administered with either ARM or BCG had less weight loss and inflammatory immunopathology in the lungs than those in the PBS control group. These pathogen clearances were associated with ARM- and BCG-boosted lung T cell activation with increasing proportion of CD44+ cells, *Mtb*-specific IFN-γ, TNF-α, IL-2, and IL-17 producing cells, serum IFN-γ, TNF-α, IL-2, and IL-10 levels, *Mtb*-specific IgG production, and the antibody-dependent H37Ra phagocytosis of macrophages and phagosome-lysosome fusion. Compared to BCG, ARM was more efficient at lowering the bacterial load than BCG, and the mice that received ARM maintained a constant body weight with a slight increase in response to treatment and had mild inflammatory immunopathology in the lungs. This superior potency was attributable to ARM induction of a higher proportion of CD44+ and cytokine-producing cells in the CD4+ T cell population than those induced by BCG. Most of those CD4+ cells produced IFN-γ and TNF-α and many of them were multifunctional, producing 3 or 2 cytokines in combination, and this elevated IFN-γ and TNF-α levels in serum of ARM-treated mice. In contrast, BCG induced mostly monofunctional with a few producing 2 cytokines. Additionally, ARM administration not only promoted ARM-specific but also *Mtb*-specific IgG level in serum whereas BCG did not promote ARM-specific IgG production. The ARM-specific IgG was more potent than other *Mtb*-specific IgG at mediating FcγR-dependent H37Ra phagocytosis of macrophages, promoting phagosome-lysosome fusion, and suppressing H37Ra replication. While both ARM and BCG exhibited immunogenicity in our model, differences in administration routes and schedules—intramuscular injection for ARM versus subcutaneous injection for BCG—may influence the magnitude and quality of the elicited immune responses, thereby confounding direct comparisons. These findings nonetheless highlight the therapeutic potential of ARM in enhancing both cellular and humoral immunity in a post-exposure context, warranting further evaluation through uniform immunization route in future studies.

T cell-mediated immunity plays a pivotal role in controlling *M. tuberculosis* infection, with CD4+ T cells primarily responsible for coordinating the immune response and producing cytokines, while CD8+ T cells contribute through cytotoxic mechanisms (67). BCG-mediated protection against TB has traditionally been attributed to vaccine-induced CD4+ T cells, which rapidly secrete Th1 cytokines and play a central role in controlling *Mtb* infection (68). This Th1-driven immune response is considered a hallmark of BCG’s protective effect. Our findings show that ARM immunization significantly enhances IFN-γ-, TNF-α-, and IL-2-producing CD4+ T cell responses. These shared effector mechanisms, including the production of key cytokines and enhanced CD4+ T cell responses, align with the immune protection observed in BCG immunization. Additionally, ARM has a significant advantage in promoting polyfunctional T cells and early cytokine responses. ARM-treated mice exhibited a significantly higher frequency of cytokine-producing CD4+ T cells in the lungs and spleen compared to both BCG- and PBS-treated mice. Importantly, ARM-induced CD4+ T cells demonstrated increased polyfunctionality, with a greater proportion of cells simultaneously expressing multiple cytokines, particularly IFN-γ and TNF-α.

This contrasts with the BCG group, where single-cytokine producers were predominant. The polyfunctional nature of ARM-induced T cells, particularly the co-expression of multiple cytokines, provides a distinctive advantage in mounting a more robust and sustained immune response (18, 19). TNF-α-producing T cells activate other defense cells, such as macrophages and dendritic cells (69). Together with IFN-γ, they stimulate the production of reactive oxygen and nitrogen species, enhancing the antituberculosis activity of macrophages (67), thereby facilitating *Mtb* elimination in ARM-treated mice. The increased frequency of IL-17A-producing T cells in the lungs of BCG and ARM groups highlights their potential to strengthen mucosal immunity, as IL-17A plays a crucial role in recruiting Th1 cells for infection control and promoting bacterial killing by neutrophils and macrophages (70, 71). Although CD8+ T cells play a relatively minor role in TB immunity compared to CD4+ T cells, they contribute to infection control through cytotoxic mechanisms and cytokine production (16, 72). Our data indicate that both BCG and ARM immunization enhance CD8+ T cell responses, with an increased proportion of total cytokines-producing CD8+ T cells compared to PBS-treated mice. Given that CD8+ T cells primarily exert cytotoxic effects via MHC class I-restricted antigen presentation (72), the induction of robust CD8+ T cell responses by ARM indicates its potential to enhance immune-mediated bacterial clearance in pre-exposed hosts. The kinetics of serum cytokine levels indicate that ARM-treated mice exhibit an earlier release of Th1 cytokines, which correlates with a more rapid reduction in tissue bacterial loads. In later stages, the gradually decreasing levels of Th1 cytokines including the IL-10, along with improved histopathological lesion and body weight recovery, suggest the resolution of inflammation and recovery from *Mtb* infection. Above all, ARM proves to be more effective than BCG in promoting a broader T cell response and earlier cytokines production, ultimately improving protection against tuberculosis. ARM’s superior performance compared to BCG may also stem from its antigenic specificity. BCG contains a broad repertoire of mycobacterial antigens, some of which have been hypothesized to act as immunological “decoys”—eliciting strong but non-protective immune responses that may impede the generation of effective host defense (73). In contrast, ARM is composed of well-defined, immunogenic components selected for their relevance to protective immunity, potentially avoiding such immune misdirection and enabling more focused immune activation.

As previously mentioned, BCG confers anti-TB immunity primarily through cellular responses (68) and only a few studies have reported that BCG vaccination can induce arabinomannan-specific antibodies, which may influence ADCP (63, 64). Our study reveals that, in addition to T cell responses, ARM immunization elicits a robust antibody response, significantly enhancing *Mtb* phagocytosis and clearance. These findings highlighting the crucial role of humoral responses in TB immunity (23, 24). Compared to BCG and PBS groups, ARM-immunized mice exhibited higher levels of ARM- and WCL-specific antibodies, reflecting both broad antigen recognition and enhanced humoral responses. ARM-induced antibodies increase opsonization of *Mtb*, marking it for enhanced recognition and uptake by phagocytic cells, further improving bacterial clearance. Notably, sera from ARM-immunized mice promoted *Mtb* ingestion and inhibited bacterial growth by increasing intracellular killing through FcγR-mediated P-L fusion, a key process for efficient *Mtb* clearance. Upon activation, FcγR signaling initiates a series of events including actin remodeling, calcium influx, and downstream kinase activation, which collectively facilitate P-L fusion and subsequent phagosome acidification (74). This acidification triggers antimicrobial mechanisms, such as reactive oxygen species (ROS) production and hydrolytic enzyme activation, thus promoting *Mtb* degradation (75), a process essential for antigen processing and presentation (76). The superior functionality of ARM-induced antibodies in facilitating FcγR-mediated P-L fusion and bacterial killing, compared to BCG-induced antibodies, may be attributed to the membrane-associated protein MPT70, as protection mediated by cell wall- and membrane-associated proteins typically occurs via ADCP (25, 63–65). These findings underscore the critical role of antibody-mediated immunity in anti-TB responses. Specifically, antibodies targeting *Mtb* outer membrane or cell wall-associated proteins, such as arabinomannan (63, 64) and PstS1 (25, 65), mediate protection via ADCP. Overall, our results demonstrate that ARM-induced antibodies significantly contribute to *Mtb* clearance and provide a mechanistic basis for ARM’s superior efficacy over BCG in both *Mtb* clearance and pathology repair. These findings underscore the potential of antibody-mediated mechanisms, including opsonization and FcγR activation, in anti-TB immunity.

Despite its promise, this study has limitations (1): The attenuated H37Ra strain was used instead of the virulent H37Rv strain due to biosafety constraints. Although H37Ra is considered a reliable surrogate under BSL2 conditions (77, 78), its reduced virulence and altered immunogenicity compared to virulent H37Rv and other clinical isolates may limit its ability to induce protective immune responses needed for human-relevant efficacy. Therefore, validation in more physiologically relevant models (79), including with virulent or hypervirulent clinical strains, is warranted (2). Our infection model used intravenous inoculation to ensure uniform bacterial delivery and synchronized systemic immune activation, enabling early assessment of cytokines responses and bacterial clearance (60). However, this route does not mimic the natural aerosol transmission of TB or the mucosal immune responses in the respiratory tract. As a result, it may underestimate the contribution of mucosal antibodies—particularly secretory IgA—which have been shown to play a protective role in pulmonary TB (27, 30, 31). Thus, future studies using aerosol challenge with virulent Mtb strains are needed to fully assess ARM’s potential to elicit mucosal immunity and respiratory protection (3). Additionally, C57BL/6 mice were used for animal experiments, a strain known for its self-limiting recovery from both H37Ra and H37Rv infection (80). This characteristic may obscure differences in vaccine efficacy during infection, necessitating validation in alternative models with sustained bacterial burdens, such as those mimicking latent infection and spontaneous or stress-induced reactivation (81) (4). The precise components of ARM-induced antibody responsible for enhancing phagocytosis remain unclear and require further study. Approaches such as epitope mapping (82) and monoclonal antibody isolation (83) can identify protective targets, while antibody depletion assays using pre-adsorbed serum (84) can pinpoint key antigens. Sequential immunizations with individual proteins and ARM boosts may clarify each component’s role. If single antigens do not reproduce ARM’s effect, a synergistic interaction among components is likely, highlighting the importance of the multivalent vaccine design. Addressing these questions will support the development of ARM as a versatile therapeutic vaccine.

In conclusion, the ARM fusion protein, evaluated as a therapeutic TB subunit vaccine in an *Mtb* pre-exposed mouse model, demonstrated superior *Mtb* clearance, antibody-mediated phagocytosis, and growth inhibition, reinforcing its potential as a promising therapeutic vaccine candidate. These findings underscore the importance of integrating both T and B cell-mediated immunity in TB vaccine design. Given the central role of chemotherapy in TB treatment, future studies should assess the integration of ARM into existing treatment regimens to enhance therapeutic efficacy and potentially shorten treatment duration. Further research is warranted to elucidate the underlying immune mechanisms, optimize vaccine formulation and delivery, and evaluate ARM’s safety and efficacy across diverse preclinical models—including its potential as a prophylactic vaccine to prevent latent TB reactivation. Collectively, these efforts will help advance ARM toward clinical application and broaden its role in TB control strategies.

## Data Availability

The original contributions presented in the study are included in the article/**Supplementary Material**. Further inquiries can be directed to the corresponding author.
