# Supplementary material for: Therapeutic vaccination with the Ag85B-Rv2660c-MPT70 fusion protein enhances Mycobacterium tuberculosis H37Ra clearance in post-exposure mice
Source: Front Immunol. 2025 Aug 14;16:1624923. doi: 10.3389/fimmu.2025.1624923 (PMC12391039; doi:10.3389/fimmu.2025.1624923)
Supplement: Supplementary file 1 [file DataSheet1.pdf]

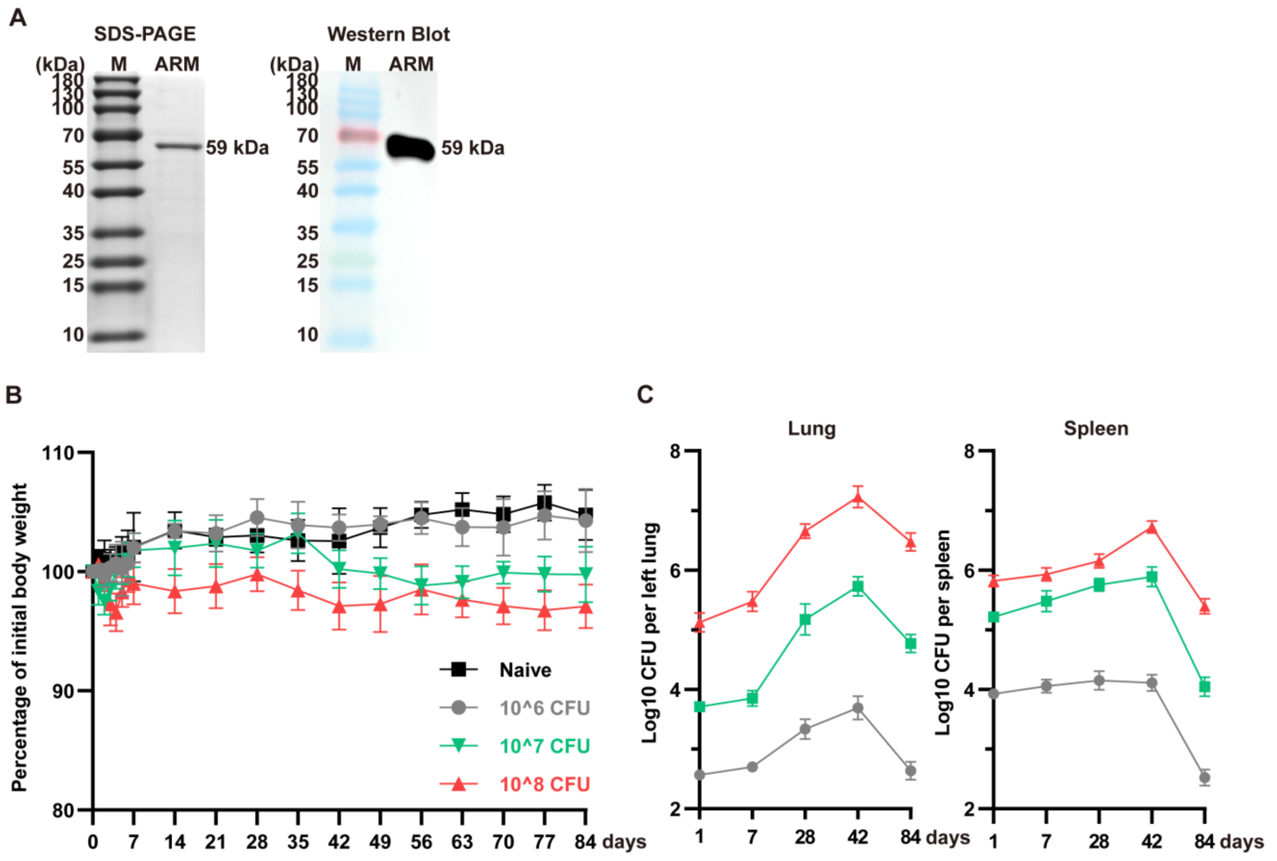

Figure S1. ARM-fusion protein purification and Mtb-exposed mice model construction. (A) Purification and identification of the ARM-fusion protein. SDS-PAGE (left) and Western blot (right) images confirm that the purity of ARM exceeds 94.5%. **(B)** Dose-response study to establish an optimized Mtb-exposure model. Body weight was recorded as a percentage of the initial weight at the time of infection. (n = 4). **(C)** Bacterial burdens in the lungs and spleen. Mtb loads in the lungs and spleen were quantified at days 1, 7, 28, 42, and 84, with CFUs presented as Log10 values. (n = 4). Data are presented as mean, with error bars representing the standard deviation (SD).

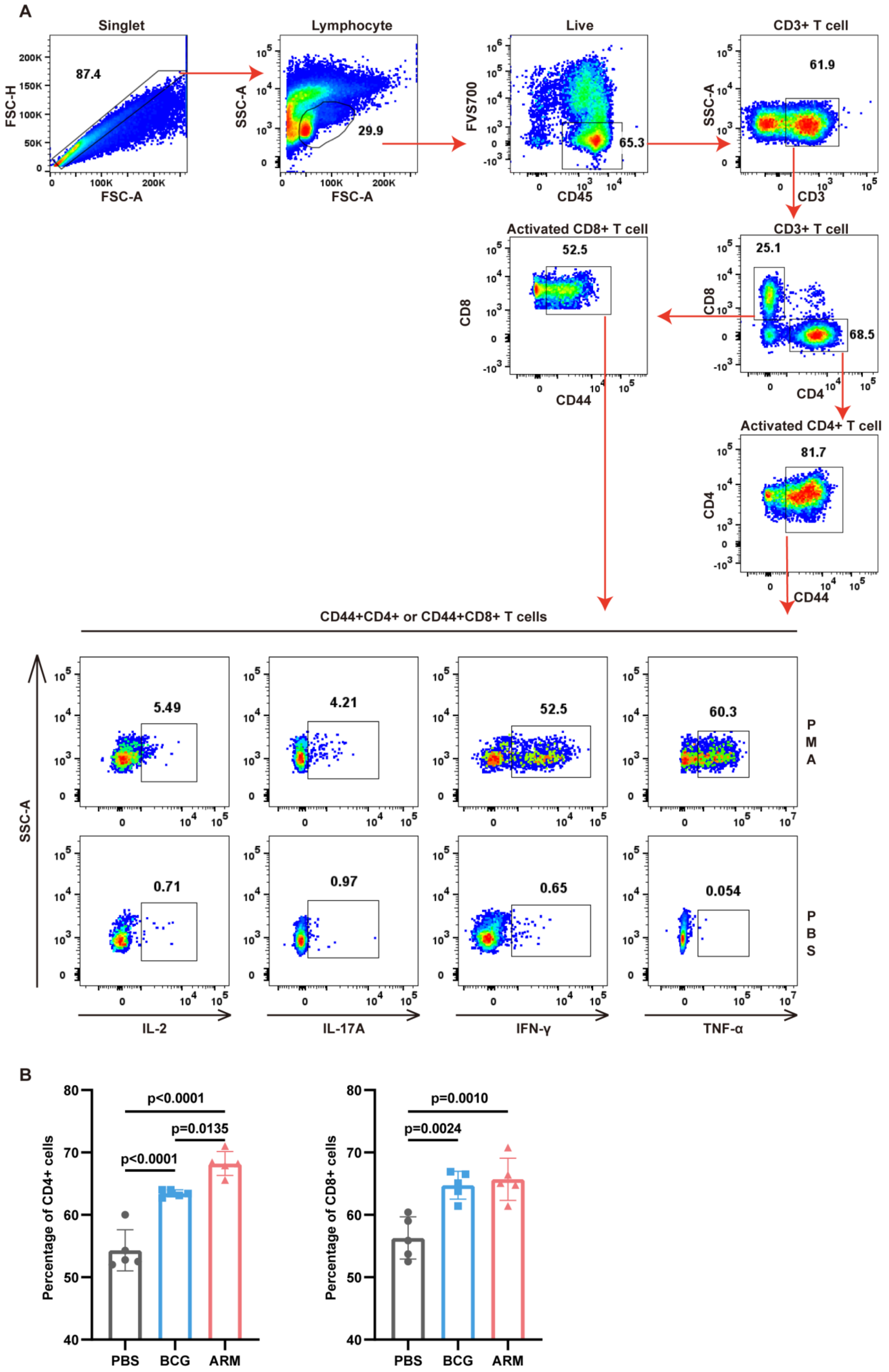
A
Singlet
87.4
FSC-H
FSC-A
Lymphocyte
29.9
SSC-A
FSC-A
Live
65.3
FVS700
CD45
CD3+ T cell
61.9
SSC-A
CD3
Activated CD8+ T cell
52.5
CD8
CD44
CD3+ T cell
25.1
68.5
CD8
CD4
Activated CD4+ T cell
81.7
CD4
CD44
CD44+CD4+ or CD44+CD8+ T cells
SSC-A
5.49
4.21
52.5
60.3
PMA
0.71
0.97
0.65
0.054
PBS
IL-2
IL-17A
IFN-γ
TNF-α
B
Percentage of CD4+ cells
p<0.0001
p=0.0135
p<0.0001
PBS
BCG
ARM
Percentage of CD8+ cells
p=0.0010
p=0.0024
PBS
BCG
ARM

Figure S2. Gating strategy for flow cytometry analysis. (A) The gating strategy for flow cytometry analysis of T cell phenotypes and cytokine-producing T cells in response to H37Rv WCL or ARM. A representative sample from an un-exposed mouse is shown, following PMA stimulation and without stimulation. (B) Flow cytometry analysis shows the percentage of CD44+ cells among total CD4+ and CD8+ T cells in the lungs of mice treated with PBS, BCG, or ARM on day 56. (n = 5 mice per group). Statistical significance was determined using one-way ANOVA, with p-values indicated. Data are presented as mean, with error bars representing the standard deviation (SD).

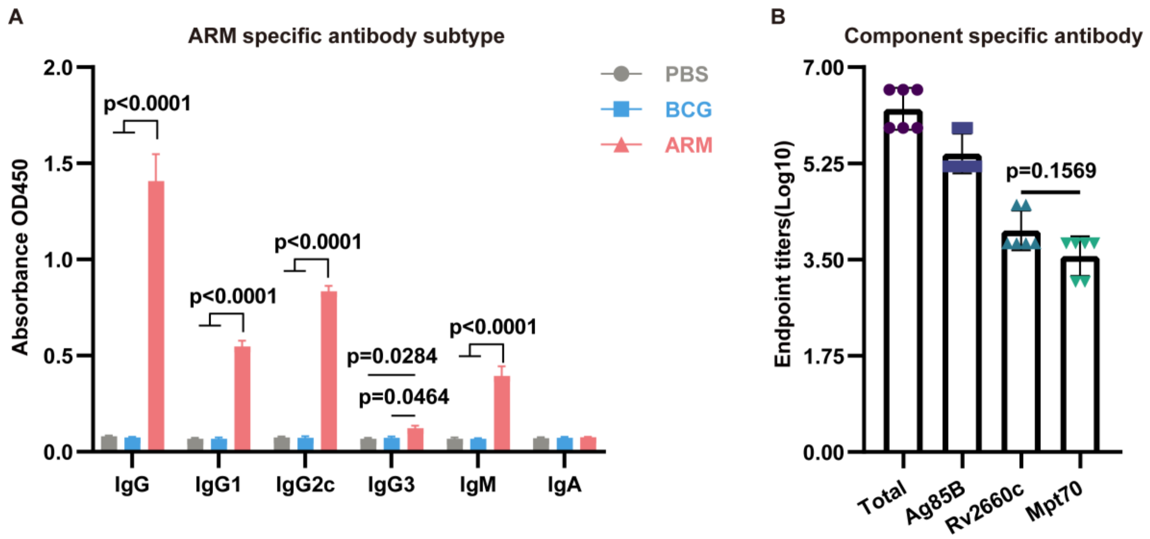

Figure S3. Subtypes identification of ARM-specific antibodies. (A) Subtypes of ARM-specific antibodies. ARM fusion proteins were immobilized on Maxisorp plates. Mouse serum from each vaccination group was used as the primary antibody, and HRP-conjugated IgG, IgG1, IgG2c, IgG3, IgM, and IgA were used as secondary antibodies. OD450 was measured to determine the antibody titers. **(B)** IgG responses to ARM single components. Ag85B, Rv2660c, or MPT70 proteins were immobilized on Maxisorp plates. Serum from ARM-immunized mice was used as the primary antibody, and HRP-conjugated IgG was used as the secondary antibody. OD450 was measured to determine the antibody titers. (n = 6). Statistical significance was determined using one-way or two-way ANOVA, with p-values indicated. Data are presented as mean, with error bars representing the standard deviation (SD).
